# Fundamental Movement/Motor Skills as an Important Component of Physical Literacy and Bridge to Physical Activity: A Scoping Review

**DOI:** 10.3390/children12101406

**Published:** 2025-10-17

**Authors:** Tomasz Piotrowski, Hubert Makaruk, Edyta Tekień, Wojciech Feleszko, Maciej Kołodziej, Katarzyna Albrecht, Krystyna Grela, Robert Makuch, Bożena Werner, Jakub S. Gąsior

**Affiliations:** 1 Department of Pediatric Cardiology and General Pediatrics, Medical University of Warsaw, 02-091 Warsaw, Poland; 2 Department of Athletics, Faculty of Physical Education and Health, Józef Piłsudski University of Physical Education in Warsaw, 21-500 Biała Podlaska, Poland; 3 Department of Pediatric Rehabilitation, Jozef Polikarp Brudzinski Public Pediatric Hospital, 02-738 Warsaw, Poland; 4 Department of Pediatric Pneumonology and Allergy, Medical University of Warsaw, 02-091 Warsaw, Poland; 5 Department of Pediatrics, Medical University of Warsaw, 02-091 Warsaw, Poland; 6 Department of Pediatric Hematology and Oncology, Medical University of Warsaw, 02-091 Warsaw, Poland; 7 Department of Pediatrics and Endocrinology, Medical University of Warsaw, 02-091 Warsaw, Poland; 8 Department of Physical Education, Faculty of Philology and Pedagogy, Casimir Pulaski University of Radom, 26-600 Radom, Poland

**Keywords:** fundamental movement skills, motor competence, physical literacy, child development, physical activity

## Abstract

Background: Movement is crucial for human development, particularly during childhood. Fundamental movement skills (FMSs) are essential movement patterns that support physical, cognitive, and social development. Recent studies indicate an alarming worldwide decline in FMS acquisition, potentially impacting children’s long-term physical fitness and health. This scoping review explored FMSs, their relationship to motor competence and physical literacy, associations with physical activity and fitness, assessment methods, and effective interventions. Methods: A comprehensive literature review was conducted using the PubMed, Web of Science, and Cumulative Index to Nursing and Allied Health Literature databases. The search utilized key phrases related to FMSs, motor competence, and physical literacy. Initially, 2251 publications were identified. Results: After rigorous screening, 95 English-language literature reviews and meta-analyses focusing on FMSs in healthy children were selected for detailed analysis. The accepted publications were categorized into five thematic areas: FMSs and motor development (11 publications), conceptual terms in FMS context (8 publications), relationships between FMSs and other parameters (15 publications), FMS assessment tools (14 publications), and intervention effects on FMSs (47 publications). Conclusions: Effective FMS acquisition requires collaborative interventions involving teachers, parents, sports professionals, and healthcare providers. Future research should focus on developing standardized assessment tools, interpreting FMSs as part of physical literacy to understand their association with PA level and design efficient intervention strategies.

## 1. Introduction

Movement constitutes an integral part of human life, playing a crucial role in physical, cognitive, and social development, particularly during childhood [1]. Achieving motor development milestones is determined in the early years by a dynamic interaction between individual characteristics—including central nervous system development and neuroplasticity [2], and genetic factors [3]—and environmental contexts encompassing both home and broader settings, task-specific demands, and the cognitive–perceptual processes underlying motor control and learning [4]. Motor development in later years is additionally dependent on regular movement practice and opportunities for exercise [1]. Experiencing diverse motor behaviors/activities between 3 and 6 years of age support the acquisition and development of fundamental movement skills (FMSs) [5,6,7]. By the age of 6 to 8 years, the majority of children have the potential to achieve proficiency in these skills [8].

FMSs are classified into three categories: (1) locomotor skills (e.g., running, jumping); (2) object control skills, which involve manipulating objects (e.g., throwing, catching, kicking); and (3) stabilization skills, which include acquiring and maintaining both static and dynamic balance [9,10]. However, the conceptualization of FMSs has been subject to considerable terminological confusion and theoretical ambiguity [11]. FMSs are defined as movement patterns that, while often building upon genetically encoded motor programs such as central pattern generators for locomotion and postural control [12,13], require refinement through appropriate teaching and practice opportunities to achieve efficient form [14]. Although primary locomotor patterns like walking and running emerge naturally through typical development as rhythmic, reciprocal movement sequences [12,13], FMSs represent more complex, goal-directed adaptations of these patterns that must be explicitly taught and practiced to reach proficiency [14]. Newell, in an article published in 2020, critically examined the prevailing classifications of FMSs, arguing that many motor skills have been inappropriately labeled as “fundamental” without adherence to the core meaning of the term [11]. He proposed that for a motor skill to be considered fundamental, it must satisfy three criteria: (i) uniqueness of the movement pattern and/or outcome, (ii) near universality of the functional outcome in the healthy population, and (iii) capacity to act as an antecedent influence supporting generalization to a large and broad set of perceptual–motor skills. Based on this framework, Newell posited that only the infant motor development sequence underpinning upright posture (e.g., sitting, bipedal standing), locomotion (e.g., walking, running), and object interaction (e.g., grasping) represents the minimum set of fundamental motor skills from which all other skills evolve across the lifespan. Skills that typically emerge during the 2- to 18-year-old range, such as throwing, catching, jumping, and hopping, while important for motor development, were proposed to be classified as “core developmental activities” rather than fundamental skills, as they exhibit greater cultural variability, lower universality in occurrence, and more restricted generalization patterns [11].

In 2018, Hulteen et al. proposed to replace the term “fundamental movement/motor skills” with “foundational movement skills”, which encompasses both traditionally understood FMSs and skills considered important in promoting physical activity (PA) across life stages (e.g., bodyweight squats, cycling, swimming) [15]. The acquisition of FMSs is considered fundamental to achieving specialized, context-specific motor skills necessary for participation in various active plays, movement games, physical activities, and sports [16], contributing to children’s cognitive and social development, as well as an active lifestyle [15].

In recent years, there has been an increasing number of studies indicating an alarming decline in FMS acquisition and development worldwide [17,18,19]. Multiple factors contribute to this decline. Cross-sectional studies demonstrate that children’s screen time is inversely related to, e.g., manual dexterity skills [20]. Opportunities for outdoor play have declined, with children increasingly engaged in structured, supervised indoor activities rather than unstructured outdoor play. Parental concerns over injury and risk, combined with increased academic/school demands and structured extracurricular activities during the after-school period, have contributed to significant reductions in time available for free outdoor play [21]. Researchers, emphasizing the strong connection between FMSs and motor competence (MC) (definition provided in Section 4) and their associations with physical fitness (PF) and physical literacy (PL) (definition provided in Section 4), suggest that children who do not acquire and/or develop their FMSs in early life may fail to develop the PF necessary for participation in various activities and sports later in life [22], and are consequently more predisposed to obesity and its consequences [23]. Considering the hypoactivity (excessively low PA levels) among children and youth prevalent in all developed countries [24] and that FMSs (a) must be learned and developed through structured and unstructured motor experiences [10,25] and (b) acquired and developed FMSs have a positive relationship with higher levels of PA [26] and PF [27], there is a growing need to implement effective intervention strategies ensuring that children acquire and develop these fundamental skills during critical life periods. The search for effective FMS interventions has been identified as one of the key research areas concerning child development in recent years [28].

The purpose of this literature review was to present the key aspects of FMSs with special emphasis on (i) the explanation of terms used in the context of FMSs: motor competence and physical literacy, (ii) the relationship between FMS acquisition and development and PA and PF, (iii) assessment tools, and (iv) interventions that may influence FMS acquisition and development.

## 2. Materials and Methods

**Figure 1:**
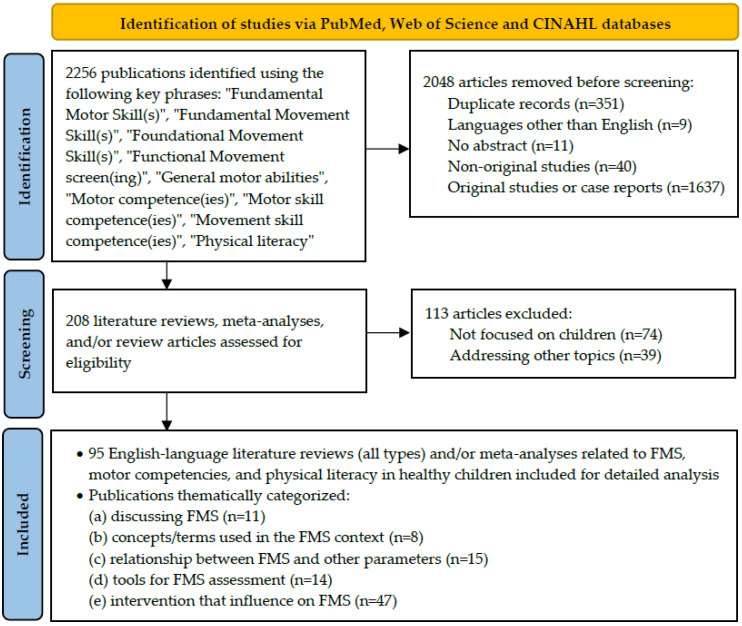
Flow diagram for the search process.

Methods were compliant with the Preferred Reporting Items for Systematic Reviews and Meta-Analyses extension for Scoping Reviews (PRISMA-ScR) checklist (Supplementary Materials). The review was conducted in accordance with the appropriate extension for a scoping review [29]. The PubMed, Web of Science, and Cumulative Index to Nursing and Allied Health Literature (CINAHL) databases were searched (articles published up to 6 December 2023) using the following key phrases in title of the study: “Fundamental Motor Skill(s)”, “Fundamental Movement Skill(s)”, “Foundational Movement Skill(s)”, “Functional Movement screen(ing)”, “General motor abilities”, “Motor competence(ies)”, “Motor skill competence(ies)”, “Movement skill competence(ies)”, and “Physical literacy”, yielding, after removing duplicates across the three databases, a total of 2256 publications. Studies were included if they were published in English, had an available abstract, were review articles (excluding commentaries, editorials, corrections, errata, study protocols, or withdrawn papers), and were not original research studies or case reports. Figure 1 illustrates the search procedure. Data from the included reviews were synthesized narratively. Key information regarding FMS definitions, associations with physical activity and fitness, assessment tools, and interventions were extracted and summarized thematically to provide a comprehensive overview of the current state of knowledge. An example of full electronic search strategy for one of the key phrases, i.e., “Fundamental Movement Skill”, is presented here: https://pubmed.ncbi.nlm.nih.gov/?term=%22Fundamental+Movement+Skill%22%5BTitle%5D&filter=dates.1000%2F1%2F1-2023%2F12%2F6&sort=date (accessed on 6 December 2023). The review protocol was not registered in any database.

## 3. Results

The following types of articles were excluded during the first stage of the screen process: 9 published in languages other than English; 11 without abstract availability; 40 classified as commentary, editorial, correction, erratum, study protocol, or withdrawn; 1637 original studies or case reports related or unrelated to the pediatric population; and 351 duplicates. After excluding publications that did not meet the inclusion criteria (through title and/or abstract analysis), a title and/or abstract analysis was performed on 208 literature reviews, meta-analyses, and/or review articles. After excluding 74 reviews not focused on children and 39 addressing other topics, 95 English-language literature reviews (all types) and/or meta-analyses related to FMSs, motor competencies, and physical literacy in healthy children were included for detailed analysis and thematically categorized (Table 1).

## 4. Discussion

World Health Organization (WHO) experts recommend daily PA and active play through diverse, enjoyable physical activities for children aged 3 to 5 years. Recommendations for older children focus on quantitative aspects—with a minimum of 60 min of moderate-intensity PA daily, along with specific PF components (i.e., resistance exercises aimed at improving muscle strength) [34]. Nevertheless, a growing number of studies document a progressive decline in PA levels among children and youth in recent years [24,35,36,37,38,39]. The WHO’s emphasis on quantity may limit considerations of qualitative aspects of PA, such as (1) motor skills and competence development, (2) socialization, and (3) exercise enjoyment [40].

Developing MC (definition—see below) is recognized as a key factor/determinant of subsequent positive health behaviors, including achieving optimal PA levels. Neglecting processes of acquisition, verification, and routine assessment of motor competencies and FMSs results in interventions targeting symptoms rather than underlying causes of suboptimal PA in children and adolescents [41].

- Concepts/Terms Used in the Context of FMSs: Motor Competence, Physical Literacy

Motor competence (MC), defined as an umbrella concept encompassing terms such as FMSs, motor ability, motor proficiency, motor performance, and motor coordination [42], refers to the degree of proficiency in executing a wide range of motor tasks and the quality of movement, coordination, and motor control [42,43]. Motor competence development depends on both biological factors (genetics, sex, maturation) and environmental factors (parenting style, stereotypes, experiences, play opportunities, encouragement, demographic) and social factors [41]. The level of positive association between MC and PA, PF, cardiorespiratory endurance, muscle strength and endurance, and negative association with sedentary behavior and body mass tends to increase from childhood to adolescence [42,43,44,45,46,47]. There is no single assessment tool that covers all aspects of motor competencies [48]. Additionally, a weak relationship has been demonstrated between actual motor competence and perceived motor competence/physical self-perception in children and youth, regardless of age, sex, developmental level, or measurement tool concordance [49]. To ensure comprehensive motor competence assessment in children, it seems reasonable to combine objective methods, such as video recording of movement using available devices and applications, with subjective methods including self-assessment and evaluation by others (e.g., parents, teachers) [48]. It appears that the success of interventions aimed at improving motor competencies [50] may not depend on their theoretical foundations, but crucially on creating an environment that motivates continuous self-improvement and skill enhancement [51]. Motor competencies developed in early childhood have a critical, significant relationship with physical literacy development.

A decade ago, physical literacy was defined as motivation, confidence, physical competence, knowledge, and understanding of the value of PA, and commitment to engagement throughout life [52]. Currently, it is emphasized as a multidimensional concept encompassing four domains: (i) physical development (motor skills, sports participation), (ii) psychosocial development (motivation, sense of confidence, self-efficacy, and effectiveness), (iii) cognitive development (knowledge and understanding of the consequences of being physically active), and (iv) integrated development [53,54,55,56,57,58,59]. Physical literacy is a child’s ability to understand their body’s movement, how to control it to consciously increase health-supporting physical or sporting activity [60]. As recently highlighted (2023), the holistic nature of this concept poses a methodological challenge in creating an appropriate measurement tool that considers children’s and adolescents’ developmental stages [30,61], consequently making it difficult to verify the effectiveness of potential interventions [62,63]. Based on studies evaluating the reliability of selected physical literacy assessment tools, the following were indicated: Physical Literacy in Children Questionnaire and Passport for Life for children, Canadian Assessment for Physical Literacy for older children, and Adolescent Physical Literacy Questionnaire for teenagers [64], although it was noted that these are not widely known [62].

- Associations of FMSs with other parameters/measures

FMSs are described as the building blocks of movement for children and youth in the process of physical literacy development [65]. An ongoing debate exists regarding which FMSs significantly facilitate current and future participation in diverse physical activities. Conclusions from successive literature reviews published over recent years are ambiguous/inconsistent in the context of the association between FMS competencies and PA levels—ranging from a lack of correlation [66,67] to a strong association [68,69]. It is possible that presenting only moderate-to-vigorous PA, rather than light-level activity, is associated with demonstrating optimal or higher levels of FMS competencies [31,70,71]. Authors of a cross-sectional analysis of eight pooled trials (age 3–11 years) using device-measured PA and standardized FMS assessment revealed that the relationship between FMSs and PA may be more complex, i.e., non-linear associations between object control skills and both moderate- and vigorous-intensity PA, with relatively weak associations in the low-to-mid ranges of skill scores, but markedly stronger associations emerging at higher proficiency levels [72]. This finding suggests that children may need to reach a threshold level of FMS competence—particularly in object control skills—before these skills translate into higher daily PA engagement. In contrast, locomotor skills demonstrated linear positive associations with PA across all skill levels. These findings indicate that the inconsistencies observed across reviews may partially stem from the use of linear statistical models that inadequately capture threshold effects, as well as from the amalgamation of different FMS domains (object control vs. locomotor) that demonstrate distinct association patterns with PA [72]. A recently published (2025) comprehensive review by Zi and de Geus of 106 systematic reviews revealed that genetic and shared environmental factors have been almost entirely overlooked in FMS–PA research [73]. The observed decline in FMS competencies and reduced PA levels may share common genetic and environmental determinants rather than representing a simple unidirectional causal pathway. This does not diminish the importance of FMS development for children’s holistic motor development and physical competence, but indicates that the relationship between FMSs and PA is more complex than traditionally conceptualized, with potential bidirectional and confounded associations that warrant consideration in intervention design [73].

Beyond assessing the relationship between FMSs and PA, researchers have also analyzed connections with other parameters—demonstrating a positive relationship between FMS competencies and cardiorespiratory endurance, and an inverse relationship between FMS competencies and body mass [68].

- Tools for Assessing Fundamental Movement Skills (FMSs)

There is no universally accepted “gold standard” for assessing FMSs. It is important to emphasize that Functional Movement ScreenTM, a leading functional assessment tool in youth athlete populations [74,75], is not dedicated to fundamental movement skills assessment. Those interested in using Functional Movement ScreenTM in children and adolescents are referred to literature reviews [76,77,78].

Existing scales, tools, and questionnaires for assessing FMSs in children and adolescents differ in approach, comprehensiveness, and psychometric properties [79]. The selection of an appropriate tool should be tailored to specific needs and conditions, considering factors such as (i) assessment purpose; (ii) participant age; (iii) availability of materials and equipment, spatial constraints affecting test feasibility; and (iv) cultural adaptations [80]. Additionally, one must remember the qualitative differences and variations in FMS acquisition between boys and girls [81]. FMS assessment tools can be categorized into two groups: (I) process-oriented tools focusing on movement quality execution, such as Test of Gross Motor Development (TGMD); and (II) product-oriented tools focusing on the end result of movement, such as Bruininks–Oseretsky Test of Motor Proficiency (BOT) and Movement Assessment Battery for Children (MABC). In most previous studies, researchers primarily used tools from the first group, with only a few employing tools from both categories [9]. Despite MABC and TGMD being among the most frequently used, they received the lowest ratings among 13 different tools in terms of applicability and feasibility for children aged 3 to 6 years. The Democritos Movement Screening Tool for pre-school children and Athletic Skills Track were rated most favorably. The analysis authors evaluated the potential applications of the examined tools while simultaneously highlighting the lack of assessment of their reliability and validity [82]. Unfortunately, a 2020 study assessing the reliability and validity of observational FMS assessment tools in school-aged children emphasized that none of the 24 analyzed tools (including MABC, TGMD, and BOT, whose psychometric properties were most frequently evaluated) meet the criteria for widespread use in schools [83].

There are more studies evaluating the reliability of FMS assessment tools than studies verifying their validity [84]. Motor skill assessment is present in over 30 tools described in the English-language literature [84]. Currently, selecting specific tests from various tools to achieve a holistic assessment of both motor task quality and its end result (performance) appears to be the optimal approach [32]. Additional implementation of technological solutions, such as inertial navigation systems using built-in mobile sensors (e.g., accelerometer, gyroscope), should provide complementary data in FMS assessment. However, these methods have significant limitations, including being costly, time-consuming, and lacking sufficient research confirming their reliability, which prevents their large-scale implementation, such as in school settings [85].

For further exploration of tools for assessing FMSs, studies by Scheuer et al. [79], Cools et al. [80], Klingberg et al. [82], Eddy et al. [83], Hulteen et al. [84], and Nagy et al. [32] offer comprehensive description (see Table 2 for details).

- Impact of Selected Interventions on Fundamental Movement Skills (FMSs)

Interventions aimed at acquiring and developing FMSs, shaping MC attitudes, and promoting PA in children can be implemented by various individuals, including the following: (a) schoolteachers, (b) parents, (c) sports club coaches, or (d) physiotherapists working with children with different disorders. The increasing hypoactivity from early childhood necessitates understanding the problem, exploring its causes, and developing diagnostic tools, including among pediatric physicians. However, it appears that collaboration among all mentioned professionals in a multidisciplinary team is crucial for achieving ultimate success [86].

In our analysis, most literature reviews related to FMSs focused on interventions affecting FMSs and/or interventions incorporating FMSs to improve PA levels. It is important to emphasize that a bidirectional relationship exists between FMSs and PA: spontaneous, unorganized PA (so-called “active play”) improved FMSs [87], while FMS training influenced PA levels [88,89]. A systematic literature review published in 2022 verified the methodological quality and effects of FMSs and PA interventions concerning cognitive and academic skills in typically developing children aged 3 to 7 years [90]. Only 6% of the included studies demonstrated high methodological quality. Combined interventions led to more favorable outcomes compared to interventions focused solely on FMSs or PA. Interestingly, interventions exclusively addressing FMSs seemed to have a greater impact on cognitive and academic skills than those focusing solely on PA [90].

- In preschool

The critical period for learning and improving FMSs is between 3 and 6 years of age [5,6,7]. In many countries, early elementary school teachers are obligated to implement physical education (PE) programs, yet they demonstrate insufficient substantive and methodological competencies and low self-confidence in conducting such classes, despite recognizing the value of PA and PE [91]. Consequently, such classes result in minimal improvement in locomotion and object manipulation skills among preschool children [92]. Preschools, where children spend several hours daily, represent environments where they can learn and develop FMSs. However, they require instructional guidance from specialists organizing time in a methodically justified manner, consistent with current didactic standards [93]. Specialized programs aimed at improving FMS development should form the basis of active time spent in preschools and early educational settings [14]. Studies have shown that teacher-led PE lessons focused on FMSs, conducted at least three times weekly, can improve FMS quality and proficiency, increase PA intensity, and reduce sedentary time among preschool children [88]. Several well-described curricular-based interventions in preschools exist, targeting single or multiple aspects: (a) teaching and improving FMSs (e.g., SKIP—Successful Kinesthetic Instruction for Preschoolers [91,92,93,94,95,96,97], The Early Steps [98]); (b) teaching FMSs and improving PA and PF levels (e.g., CHAMP—Children’s Health Activity Motor Program [99]); and (c) teaching FMSs, improving PA and PF, providing nutritional education, and parent education (e.g., Active Play [100]). A 2021 literature review emphasized the effectiveness of these programs, noting that intervention effects depend on the teacher, teaching strategies, and children’s socioeconomic status [16]. For teachers, the most significant barriers to implementing structured PA were opportunities (such as other commitments, priorities, or spatial limitations in early education centers) and capabilities (lack of PA and PE knowledge, absence of direct practical skills). Importantly, we currently know little about factors influencing increased teacher motivation for education in programs aimed at improving children’s PA [33]. However, it is known that educating parents/guardians about FMSs, involving them in their children’s FMS acquisition and improvement process, and empowering them as PA educators seem fundamental for FMS development, especially in young children [101].

- At school

Participation in PE lessons influences improvement across multiple health domains in school-aged children and youth—with the most convincing evidence showing impacts on physical health, particularly increased PA levels, improved cardiorespiratory fitness, and body mass reduction [102]. There are numerous original studies, as evidenced by the number of literature reviews, examining the relationship between PE lesson quality and topic with FMS competence levels in school-aged children. Optimized PE lessons in terms of quality (focused on implementing teaching strategies or enriched with elements aimed at fitness improvement) were associated with only minimal improvements in PF and FMSs, regardless of lesson frequency or duration [103]. Participation in school-based exergaming (active video game) sessions allowed for improvements in balance [104] and locomotion [105], but without unambiguous results in object control [104]. It appears that game type/specificity and intervention duration may be key determinants for achieving better outcomes—games that encourage or enforce FMS exercises [106], as well as supervised exergaming participation lasting over 18 weeks, enabled FMS improvement and enhancement of selected PF parameters in children and youth aged 3 to 15 years [106,107].

Physical education lessons incorporating progressive strength training can have positive effects [108]. Resistance training in children from 5 years of age demonstrates a positive impact on multiple FMSs assessed using product-oriented tools [109]. School-based neuromuscular training programs have been shown to produce greater improvements in FMSs, postural control, and muscle strength compared to standard PE lessons. To achieve optimal adaptation, the “dose” of muscle strength, stability, plyometrics, and object manipulation training should be determined considering the child’s growth and development [110]. Multi-component programs [111] combining (a) PE lessons, conducted by physical educators who acknowledge how experience of PE impacts children motivation to continue PA beyond school [112], incorporating (i) implicit and explicit motor learning methods [113], (ii) strategies that focus on all principles and components of PL [114], (iii) neuromuscular training focused on FMSs improvement with (b) mathematics, geography, or foreign language lessons that include movement-based learning (learning through movement) in school-aged children [115] may represent an optimal solution to address the currently observed global child hypoactivity. Intensive PE lessons and lessons from other subjects incorporating movement elements can reduce risky activities children undertake during class breaks. Recent research (2022) demonstrated that children derive satisfaction from taking risks and challenges, expressing a desire for greater freedom and a broader range of play opportunities during school breaks [116].

Sports activities effectively promote FMS development [117]. As an example, participation in soccer training, which requires players to possess well-developed physical, psychological, technical, and tactical skills [118], influences improvements in running speed, jump height, and object control. The effects are more pronounced in children aged 7 to 9 years compared to those training between 10 and 13 years old. Regardless of age, the effects are better for those training for a longer duration [119]. Injury prevention programs, composed of at least three components from the following: strength exercises, exercises targeting mobility, balance and/or agility, and plyometric exercises, such as FIFA 11+ Kids [120], have a positive impact on biomechanical parameters and neuromuscular performance across three FMS categories. Meta-analysis authors demonstrated a positive effect size for jump height, running speed and acceleration, and dynamic balance. Implementing components from injury prevention programs into PE lesson plans in schools may positively influence FMS refinement [121]. Similar to soccer participation, regular involvement in swimming training positively affects FMS development [122].

- The role of teachers, the role of parents

Undoubtedly, early elementary and PE teachers in primary schools are capable of conducting lessons that enable children to progress in FMSs [123]. However, the role of teacher education quality in this process remains unclear. Continuous training and teacher support appear crucial for implementing effective PE programs aimed at FMS acquisition and development, improving motor competencies, PA levels, and children’s physical literacy [124,125,126]. Interventions conducted by educated specialists—PE teachers familiar with FMSs, supported by additional home-based practice implemented by engaged parents—proved more effective in improving FMS performance than school PE classes alone [127].

The quality and context of PE lessons are as important as their structure—in other words, it is not just about what the pedagogue does, but also how and where they do it [128]. Educated, creative PE teachers aiming to improve FMSs, motor competencies, and PA levels in children and youth should consider implementing environmental variability in lessons. It is crucial that FMSs are developed in diverse environments [129,130]. One must remember that specific environments—home, school, sports hall—can both stimulate or limit motor behaviors [131]. Changing the location of PE or active play lessons from indoor spaces to outdoor environments is significant for shaping identity and relationships with others [132,133]. Activity in natural environments promotes a sense of freedom, joy, and curiosity, significantly contributing to child autonomy development. Conscious selection of locations for outdoor physical activities is necessary in both educational and health contexts [133]. For example, green space exposure between birth and hospitalization for bronchiolitis was associated with lower asthma risk in children up to 6 years old [134]. Unstructured or child-initiated outdoor activities enable diverse environmental interactions. Materials commonly referred to as “loose parts” stimulate child-directed play. However, unambiguous results are lacking regarding potential mechanisms supporting active outdoor play and FMS development [135]. Assessing relationships between early childhood education based on nature contact, PA levels, FMSs, and health requires further research [136].

- Intervention Dosage

Physical activity interventions are ambiguously reported in the context of various medical conditions. If we adopt the paradigm that physical exercises are a type of medication, then they should have a specified dosage [137]. This would allow for building a scientific argument regarding the effective “dose” in practice [128]. As evident from the presented literature review scope concerning FMSs in children, there are >95 literature reviews on this topic. Nevertheless, an algorithmic approach remains to be established that would provide guidelines for research tools and intervention dosage selection influencing the acquisition and refinement of FMSs, consequently improving PF, activity levels, and physical literacy. The application of FITT (frequency, intensity, time duration, type of exercise) [129], SAAFE (Supportive, Active, Autonomous, Fair, Enjoyable) [138], and TARGET (Task, Authority, Recognition, Grouping, Evaluation, and Time) [139] principles in future intervention studies related to FMSs may contribute to developing practical guidelines that assist in planning, implementing, and evaluating interventions supporting FMSs for children and youth. As an example, Grace et al. conducted a study at a medical center where 30 pediatric residents received training on the FITT principle through lectures, curriculum content, and an electronic smart-phrase tool to improve PA counseling for children aged 0–19 years in primary care settings. Chart reviews of 423 patient encounters over 16 months showed significantly increased documentation of PA frequency post-intervention, rising from 31.9% to 50.9%, although no significant changes were observed in documentation of intensity, time, or type components [140]. The ATLAS (Active Teen Leaders Avoiding Screen-time) obesity prevention program was conducted in 14 secondary schools located in low-income communities, involving adolescent boys aged 12–14 years who were at risk of obesity. Teachers were trained to deliver enhanced sport sessions using SAAFE teaching principles, which were specifically designed to enhance students’ autonomous motivation for physical activity. The ATLAS intervention included professional development, fitness equipment for schools, teacher-delivered physical activity sessions, lunch-time activity sessions, researcher-led seminars, a smartphone application, and parental engagement strategies. Assessments for primary outcomes (body mass index and waist circumference) and secondary outcomes were conducted at baseline, 8 months post-intervention, and 18-month follow-up to evaluate the sustained impact of the program [141].

- The role of pediatric physicians, physiotherapists and occupational therapists

Children presenting with delayed psychomotor development [142,143], premature birth [144], and pediatric patients with neurological [145], pulmonological [146,147], or cardiac disorders [148], those post-liver transplantation [149], and oncological patients during and after cancer treatment [150,151] all demonstrate delays in acquiring and/or deficits in executing FMSs.

One of the objectives in the 2030 Sustainable Development Agenda is to ensure high-quality early childhood development access for all girls and boys by 2030 [152,153]. Pediatric physicians should be familiar with the topic of widespread hypoactivity among children and the concept of FMSs. This knowledge will enable them to assess PA and refer patients to physiotherapists, rehabilitation specialists, and strength and conditioning specialists to assess physical development and determine the necessity of implementing optimal interventions.

Pediatricians can simply assess PA using brief, validated screening tools integrated into routine clinical visits. The Physical Activity Vital Sign is recommended—a two-question assessment asking about the number of days per week a child achieves 60 min of moderate-to-vigorous PA and the types of activities they engage in (such as active transportation, physical education, organized sports, and screen time). Results can be incorporated into electronic health records to facilitate systematic assessment at health supervision visits for children ages 6–18 years, with the goal of identifying insufficient activity and guiding tailored counseling [141,154].

Physiotherapists and/or occupational therapists providing care for pediatric patients aged 3 to 6 years should, even if not the primary consultation purpose, assess FMSs. When formulating therapeutic goals, it is important to remember the “F-words” paradigm [155], which is compatible with the International Classification of Functioning, Disability and Health [156]. According to the “F-words” concept, therapeutic goals should be established at the following levels: structure and function (fitness), activity (functioning), participation (friends). These goals should also consider environmental factors (family), individual factors (fun), and child development and maturation (future) [156]. Acquiring and/or developing FMSs in a pediatric patient aged 3 to 6 years could be defined as a therapeutic goal at the activity level. In this context, one would need to determine which elements at the structure and function level (e.g., muscle strength, PF), participation level (e.g., teacher’s role), environmental factors (e.g., parental role), and individual factors (e.g., child’s temperament, motivation, and willingness) require improvement.

The interconnected nature of these elements requires interventions that address the complex web of factors influencing PA behavior rather than isolated components. At the structure and function level, muscular strength (F-words → Force) serves as a foundational element for functional movement competence, with the relationship between FMS proficiency and PF strengthening progressively from early childhood to adulthood [157]. Children with poor movement skill proficiency may struggle to break through a motor proficiency barrier that limits engagement in age-appropriate activities [157]. The quality of movement experience—not merely quantity—plays a crucial role in sustaining engagement. Interventions must be enjoyable (F-words → Fun), developmentally appropriate, and personalized to foster intrinsic motivation [157]. The “My Physical Activity Plate” framework prosed by Faigenbaum and co-workers very recently (2025) emphasizes variety, flexibility, and personal choice, allowing children to select activities based on preferences and capabilities, thereby reducing intimidation barriers and making PA more achievable [158]. Environmental factors significantly influence early childhood activity patterns. Children whose parents serve as active role models and who play outdoors with family are more likely to meet recommended PA levels [157]. Access to safe facilities and qualified facilitators who can design developmentally appropriate programs is essential for addressing neuromuscular limitations and overcoming barriers [157]. By starting with manageable sessions that build confidence and competence, inactive children can develop self-efficacy and establish lasting habits while respecting individual autonomy and preferences [158].

**Figure 2:**
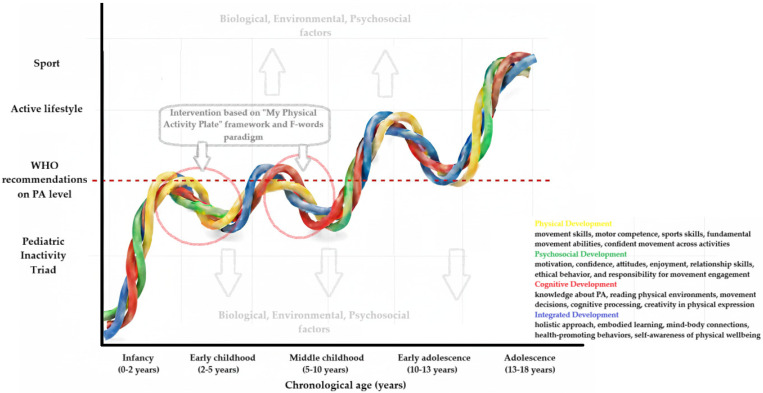
Interplay between physical literacy and physical activity in pediatric populations.

The ultimate aim is to propose an intervention that can be maximally effective—improve physical literacy and consequently PA level for the population of healthy, but inactive children and pediatric patients [159]. Figure 2 presents our understanding of association between PL and PA—a sinusoidal curve composed of four helical lines illustrating physical, psychosocial, cognitive, and integrated development (four meta-themes of PL [58]) that is facilitated or compromised by biological, environmental, and psychosocial factors. The presence of at least one, but typically interconnected conditions, from the Pediatric Inactivity Triad [39] i.e.,: physical Illiteracy (insufficient motor skill competence, confidence, or motivation to participate effectively in fundamental movement activities); exercise deficit disorder (failing to accumulate the recommended 60 min of moderate-to-vigorous PA daily); and/or pediatric dynapenia (measurably low muscular strength and power relative to age-appropriate norms without underlying neuromuscular pathology) warrants clinical attention and intervention [157,158] to achieve desirable PA level and active lifestyle [34].

- Practical recommendations for clinicians:

1. Routine screening for early identification of FMS deficiency:
  ○ Collect information about PA patterns, play opportunities, and previous motor development milestones.
  ○ Particularly between the ages of 3 and 6—ask about a child’s ability to run, jump, throw, catch, and keep static and dynamic balance.
2. Prescribing intervention:
  ○ In the presence of FMS deficits, children should be referred to pediatric physiotherapists who should conduct screening tests (e.g., TGMD).
  ○ In patients with chronic diseases, individualized PA plans incorporating FMS elements are recommended.
3. Parental education:
  ○ Educate caregivers about the importance of movement quality—coordination, balance, and skill acquisition—over simple metrics such as step count or minutes of activity.
  ○ Encourage home-based play and structured activities that develop motor skills.

## 5. Conclusions

Fundamental movement skills are critical to child development, facing significant challenges in the current global context. The research reveals a concerning decline in children’s movement skill acquisition, with potential long-term implications for physical health and cognitive development. The critical period for FMS development occurs between 3 and 6 years of age, requiring comprehensive, multidisciplinary interventions. Key stakeholders—teachers, parents, and coaches—play crucial roles in supporting skill development; however, health professionals should be integrated into multidisciplinary teams for the promotion of FMSs. To fill the gaps in existing research (lack of validated tools, scarcity of longitudinal studies), future research should focus on developing standardized assessment methods, understanding optimal intervention strategies, and addressing the global decline in children’s physical movement skills. The ultimate goal is to create supportive environments that promote lifelong physical literacy and active, healthy lifestyles.

## Figures and Tables

**Table 1:** Most recent papers accepted for detailed analysis according to thematical category.

Category (Number of Accepted Articles for Detailed Analysis)	Most Recent Paper Included for Detailed Analysis
Discussing FMSs and motor development (N = 11)	Wang JW, et al. Global hotspots and trends in research on preschool children’s motor development from 2012 to 2022: a bibliometric analysis [28].
Concepts/terms used in the FMS context: motor competence, physical literacy (N = 8)	Grauduszus M, et al. Definitions and assessments of physical literacy among children and youth: a scoping review [30].
Relationship between FMSs and other parameters (N = 15)	Liu Y, et al. The Bidirectional Correlation between Fundamental Motor Skill and Moderate-to-Vigorous Physical Activities: A Systematic Review and Meta-Analysis [31].
Tools for FMS assessment (N = 14)	Nagy ÁV, et al. Assessment Tools Measuring Fundamental Movement Skills of Primary School Children: A Narrative Review in Methodological Perspective [32].
Intervention effects on FMSs (N = 47)	Jerebine A, et al. Educator-Perceived Barriers and Facilitators to Structured-Physical Activity in Early Childhood Centres: A Systematic Review [33].

**Table 2:** Selected review articles on tools for assessing FMSs.

First Author and Reference	Article Offers Complementary Perspectives on:
Scheuer et al. [79]	Motor testing instruments used in primary school settings by reviewing and synthesizing their theoretical frameworks, psychometric properties, and areas of application, while highlighting the distinctions between tests based on motor abilities, motor skills, and motor competencies constructs
Cools et al. [80]	Movement skill assessment tools by comparing seven standardized instruments used for evaluating FMSs in typically developing preschool children, examining their content validity, reliability, administrative feasibility, and normative data quality across European and international contexts
Klingberg et al. [82]	The feasibility of 13 FMS assessment tools available for use with pre-school aged children (3–6 years), providing a systematic comparison of considerations such as administration time, equipment requirements, space needs, training demands, and qualification requirements
Eddy et al. [83]	The validity and reliability of 24 observational FMS assessment tools for school-aged children by examining their psychometric properties through the COSMIN framework to evaluate their suitability for universal screening in educational settings
Hulteen et al. [84]	The validity and reliability of 57 motor competence assessments in children and adolescents providing complementary perspectives on (1) measurement properties across diverse populations—examining both child (3–12 years) and adolescent (13–17 years) populations; (2) multiple assessment approaches—including process-oriented, product-oriented, and hybrid assessments, as well as single-skill and battery assessments; (3) comprehensive measurement properties—examining content validity, construct validity, criterion validity, internal consistency, test-retest reliability, intra-rater reliability, and inter-rater reliability; and (4) assessment content and prevalence
Nagy et al. [32]	FMS assessment tools by analyzing them from a methodological perspective, examining their test items, tools, time requirements, and the specific types of FMSs they measure, rather than focusing solely on psychometric properties like validity and reliability

## Data Availability

Not applicable.

## Supplementary Materials

The following supporting information can be downloaded at https://www.mdpi.com/article/10.3390/children12101406/s1: File S1: Preferred Reporting Items for Systematic Reviews and Meta-Analyses extension for Scoping Reviews (PRISMA-ScR) checklist.

## Abbreviations

The following abbreviations are used in this manuscript:

FMSs	fundamental movement skills
PA	physical activity
MC	motor competence
PF	physical fitness
PL	physical literacy
WHO	World Health Organization
TGMD	Test of Gross Motor Development
BOT	Bruininks–Oseretsky Test of Motor Proficiency
MABC	Movement Assessment Battery for Children
PE	physical education
ICF	International Classification of Functioning, Disability and Health
